# Non-human contributions to personality neuroscience – from fish through primates. An introduction to the special issue

**DOI:** 10.1017/pen.2022.4

**Published:** 2022-09-20

**Authors:** Yury V. Lages, Neil McNaughton

**Affiliations:** 1 Department of Psychology, Pontifical Catholic University of Rio de Janeiro, Rio de Janeiro, Brazil; 2 Department of Psychology, University of Otago, Dunedin, New Zealand

**Keywords:** Evolution, Personality, Psychopathology, Neuroscience, Translation

## Abstract

The most fundamental emotional systems that show trait control are evolutionarily old and extensively conserved. Psychology in general has benefited from non-human neuroscience and from the analytical simplicity of behaviour in those with simpler nervous systems. It has been argued that integration between personality, psychopathology, and neuroscience is particularly promising if we are to understand the neurobiology of human experience. Here, we provide some general arguments for a non-human approach being at least as productive in relation to personality, psychopathology, and their interface. Some early personality theories were directly linked to psychopathology (e.g., Eysenck, Panksepp, and Cloninger). They shared a common interest in brain systems that naturally led to the use of non-human data; behavioural, neural, and pharmacological. In Eysenck’s case, this also led to the selective breeding, at the Maudsley Institute, of emotionally reactive and non-reactive strains of rat as models of trait neuroticism or trait emotionality. Dimensional personality research and categorical approaches to clinical disorder then drifted apart from each other, from neuropsychology, and from non-human data. Recently, the conceptualizations of both healthy personality and psychopathology have moved towards a common hierarchical trait perspective. Indeed, the proposed two sets of trait dimensions appear similar and may even be eventually the same. We provide, here, an introduction to this special issue of *Personality Neuroscience*, where the authors provide overviews of detailed areas where non-human data inform human personality and its psychopathology or provide explicit models for translation to human neuroscience. Once all the papers in the issue have appeared, we will also provide a concluding summary of them.

This paper provides background for focussed reviews that will make up a Special Issue, *Non-human contributions to personality neuroscience – from fish through primates*. It also invites pre-submission enquiries.^1^

The Special Issue aims to make clear: (1) that non-human work of all types allows comparative analysis (from fish through primates) important for theories of personality in general and personality neuroscience in particular; (2) how strain derivation and neural manipulations generate non-human results that inform traits, particularly those of interest in human psychopathology (where Eysenck’s 3-factor model is still held in high regard, albeit with a need to rename his factors); (3) that observational non-human work, particularly in primates, can link to and inform the Big 5, HEXACO, etc; (4) that the different forms of non-human work can be naturally linked through study of the conserved brain systems involved – and so provide a basis for the integration of current hierarchical trait models of psychopathology (e.g., MMPI and HiTOP) with hierarchical trait models of healthy personality; (5) that, particularly between species, neural variation can help us link personality to brain systems. In sum, the Special Issue aims to show that, because of phylogenetic conservation of fundamental traits, even organisms as simple as fish can provide an architectural bedrock on which we can progressively build our understanding of the more elaborate superstructures on which personality depends in more complex organisms.

We believe that direct contact with neurobiology, both for derivation of measures and their validation (see Section 6), is crucial for more mechanistic, explanatory, theory in personality research. “Personality is an abstraction used to explain consistency and coherency in an individual’s pattern of affects, cognitions, desires and behaviors. … The task of the personality researcher is to identify the consistencies and differences within and between individuals … *and … to explain them*” (Revelle, 2007, p. 37, our emphasis). Where the explanation is neural, all current theories must align with a single set of known neuropsychological facts – with the brain (and phylogenetically conserved functions) providing a Rosetta stone to translate between the theoretical systems. Neurobiology should help us unite the Big-5, HiTOP, and Eysenck/Gray/RST approaches. Since these approaches originate in different top-down and bottom-up perspectives, integrating them across all the different motivational brain networks and levels of explanation should hit home in the heartlands of mainstream personality psychology. But first, we provide some background to this approach.

## 1. Darwin and the conservation of emotions

“On the Origin of Species by Means of Natural Selection” (Darwin, 1859) focused on non-human animals and plants to reduce opposition. It, nonetheless, implied that humans had been subject to natural selection. Ten years later, “The Descent of Man” and “Expression of the emotions in man and animals” (Darwin, 1871, 1872) treated humans as just another animal – with evolved, often phylogenetically conserved, emotions as well as morphology.

Based on his observation of facial expressions in humans, Darwin identified a few core emotions (e.g., happiness, sadness, fear, and surprise) that would have common features across cultures (Snyder, Kaufman, Harrison, & Maruff, 2010) and be based on emotional systems that are evolutionarily old and largely conserved. The importance of facial expressions for social communication in primates (Altschul, Robinson, Coleman, Capitanio, & Wilson, 2019; Wilson et al., 2020) is consistent with Darwin’s original hypothesis.

“Emotion” clearly encompasses states of affect, behaviour, cognition, and desire that sustain life using fundamental “survival circuits” (Ekman, 1992; Ledoux, 2012). However, “What is an emotion?” (James, 1884) is still answered in different ways by different people, and we have argued (McNaughton, 1989) that an emotion is most easily characterized by the “goals” (“teleonomy”, Pittendrigh, 1958) of its phylogenetic history.

If a change in state is adaptive, trait sensitivity must also depend on adaptive value (Blanchard & Blanchard, 1989). The long-term trait control of emotions and its linkage to neurological and psychiatric illness (Greene et al., 2020; McNaughton, 2020) make non-human models of emotional behaviour a valuable platform to study the conserved fundamental states and traits contributing to human emotions. According to Darwin, comparative work is less “liable to confound conventional or artificial gestures and expressions with those which are innate or universal” (Darwin, 1872, p. 50).

## 2. Conservation of brain systems

If trait patterns of emotion-related behaviour are conserved, so must be their brain mechanisms, which will be central to understanding the neural basis of personality. Subcortical structures are the primary responders to, *and organisers of*, responses to emotionally relevant stimuli (Barrett, 2017; Ledoux, 1991, 1996; Lopes da Silva, Witter, Boeijinga, & Lohman, 1990; MacLean, 1949, 1952). Thus, the subcortex is where we must first look for the long-term sensitivities that underlie personality; it is also important for cognition (Janacsek et al., 2022).

**Figure 1.**
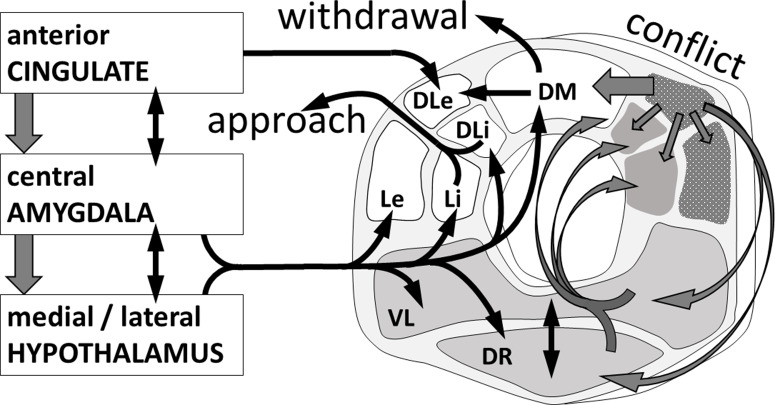
The organization of goal control within the PAG/DR and in relation to hierarchically organized afferents. From Silva and McNaughton (2019) with permission.

The periaqueductal grey (PAG) is the lowest level of the *integrated* control of emotions and has highly conserved structure and gene and protein expression across vertebrates (O’Connell & Hofmann, 2012). The PAG, hypothalamus, and amygdala are inter-connected in ancient systems that provide the most basic organised control of responses directed to appetitive and aversive goals, and to conflicts between appetite and aversion – with each of these 3 types of process controlled by a different part of the PAG (Figure 1). Posterior/dorsal PAG organises basic aversion, anterior/lateral PAG organises appetite and courtship (Comoli, Ribeiro-Barbosa, & Canteras, 2003; Kyuhou & Gemba, 1998; Mota-Ortiz, Sukikara, Felicio, & Canteras, 2009); and dorsolateral PAG and dorsal raphe organise responses to conflict between positive and negative goals (Figure 1). Separate PAG areas control active versus passive coping strategies (Keay & Bandler, 2015).

From PAG, through hypothalamus, to amygdala, neural control is well conserved relative to basal vertebrates. The PAG also receives descending input from the habenula, which is essentially unchanged from the lamprey through vertebrates (Loonen & Ivanova, 2015, 2016), despite involvement in many higher order processes (Hones & Mizumori, 2022; Loonen & Ivanova, 2019; Rolls, 2017). Further, “the habenula … plays an essential role in regulating the intensity of reward-seeking and adversity-avoiding behavior … by regulating the activity of ascending midbrain monoaminergic tracts” (Loonen & Ivanova, 2019, p. 233), which are also highly conserved with their diffuse collateral projections retained as the telencephalon expands. In zebrafish, responses to predictive and factual threats involve brain structures that, despite inverted morphology,^2^ control the same responses as in humans. These zebrafish reactions can be linked to anxiety (Mathuru & Jesuthasan, 2013). Likewise, the zebrafish can be used as a “reduced” model of a range of human emotional and cognitive disorders (de Abreu et al., 2020; Fontana et al., 2019; Gerlai, 2020; Soares, Gerlai & Maximino, 2018) .

PAG is a key structure for emotion generation. Even after hypothalamic and amygdala lesions, electrical stimulation of posterior/dorsal PAG in rats elicits escape reactions in the absence of external threat (de Molina & Hunsperger, 1962; Schreiner & Kling, 1953) – producing panic-like responses (Ballesteros, de Oliveira Galvão, Maisonette, & Landeira-Fernandez, 2014). This panic response to PAG stimulation is conserved in humans (Del-Ben & Graeff, 2009) and, similarly, depressed patients show irregular PAG functional connectivity (Truini et al., 2016).

PAG remains involved with more complex emotions. In healthy humans, social rejection increases activity in the dorsal anterior cingulate, amygdala, *and* PAG (Eisenberger, Gable, & Lieberman, 2007). In this hierarchy, higher levels control response production by interacting with the lower (Figure 1). Clearly, the PAG is where we should look for a neural sensitivity that gives rise to a panic-prone facet of personality or for basic panic psychopathology (that would couple with neuroticism to deliver panic disorder).

Above the PAG are the hypothalamus (archetypally associated with detailed motivational control), and the amygdala (Figure 1). The amygdala is complex, spans the subcortex and cortex, and is critical for the control of arousal with all motivations (Murray, 2007). Roughly one third of its neurons respond selectively to motivationally relevant stimuli in primates (Fuster & Uyeda, 1971). In all mammals, cortex and subcortex send positive and negative valence signals that the amygdala integrates to elicit adaptive behaviours via downstream targets (Correia & Goosens, 2016; McDonald, 1998; Smith & Torregrossa, 2021; Stefanacci & Amaral, 2002). Disruption in valence encoding is linked to the development of mood disorders in non-human models (Perusini & Fanselow, 2015) and humans (Brock, Harp, & Neta, 2022; Sequeira, Forbes, Hanson, & Silk, 2022).

In sum, fundamental aspects of emotional traits and of psychopathology are controlled in humans by conserved systems including diffuse ascending components that terminate throughout the neocortex (Dubois, Galdi, Han, Paul, & Adolphs, 2018; Dubois et al., 2020). This allows non-humans, from fish through primates, to provide meaningful models (with true homologies) of the core emotion production systems through which the complex sensory filters of more recently evolved cortical systems (Falcone et al., 2020; Miller, Hof, Sherwood, & Hopkins, 2021) change affect, behaviour, cognition, and desire. Both between and within species, trait aspects of these systems depend on genes and their interaction with the developmental environment of the organism. Here, in particular, non-human models are useful.

## 3. Genes, environment, and personality

Genes are a scaffold that constrains the external factors that mould emotion-processing circuits and so shape personality and psychopathology. Parental socioeconomic status, parenting practices, peer relationships, romantic relationships, and work experiences all affect personality traits (Ayoub & Roberts, 2017) and their stability into adulthood (Hopwood et al., 2011; Roberts & DelVecchio, 2000). Early-life adversity is a strong determinant of maladaptive personality in adults (de Carvalho et al., 2015; Perna, Vanni, Di Chiaro, Cavedini, & Caldirola, 2014; Rademaker, Vermetten, Geuze, Muilwijk, & Kleber, 2008; Schouw, Verkes, Schene & Schellekens, 2020). These trait effects depend on physiological alterations that include epigenetic modulation (Alshaya, 2022), HPA axis dysfunctionality (Lopez et al., 2021), and interruption of normal brain development (Marshall, Fox, & Group, 2004). Neither such environmental effects, related genes, nor their interaction can be thoroughly studied experimentally in humans. Here, non-human models are an important tool.

For example, chronic stress is thought to interact with genes to generate mood disorders in humans. “Carioca” rats, selectively bred to have high or low inherent anxiety responses allow us to assess the direction of the stress-anxiety association. Chronic unpredictable mild stress during development produces a greater increase in later reactions to threat in the high responding rats than in the low (Lages et al., 2021).

Environmental effects can also be studied in depth. For example, maternal separation in rodents and primates produces effects homologous to separation in humans. Macaques show that social factors are important (Kaufman & Rosenblum, 1969). Pigtail macaques live in small groups and their infants show strong separation reactions, easily characterised as grief and severe depression. Bonnet macaques cluster in larger groups and their infants’ separation reaction, rather than strong distress, is to interact with other adults, generating solicitous behaviours including adoption. Rodents show that early adversity leads later in life to anxiety-like behaviours and increased stress responsiveness (Hegde & Mitra, 2020) in a sex- and age-dependent manner (Réus et al., 2021; Zanta, Suchecki, & Girardi, 2021) that can be enhanced by acute stress (Zanta et al., 2021) and ameliorated by an enriched environment (Réus et al., 2021).

Importantly for personality neuroscience, the amygdala, hippocampus, and endocrine system are substrates of these responses to early adversity (Ellis & Honeycutt, 2021; Qin et al., 2021) with similar brain changes apparent in humans (Pollok et al., 2022). The comparison of strain selection and environmental experiments in non-humans with confirmatory, albeit correlational, human imaging is a powerful solution to the problems facing each approach separately.

## 4. Cognition and personality

But what of more complex cognitive processes? While subcortex is substantially conserved between basal insectivores and humans, and archicortex (hippocampus) retains its basic structure and expands only moderately (×4), neocortex is greatly expanded (×150) and elaborated (Stephan & Andy, 1969). How far can rodent neocortex (and traits it controls) be seen as homologous to human?

The cortical elaboration of basic emotional traits operates to some extent through, and retains much of, primordial emotion control (the expansion of isocortex is functionally peripheral, with older allocortex in the deeper functional zones). Phylogeny layers fine-grain facet detail onto this primordium; elaborating species-specific expression of the same fundamental phenomena. Different triggers (via different sensory modalities and schemae) support innate simple phobias: the mouse fears the (smell of the) rat; the rat fears the (smell of the) cat; the human fears the (number of legs of the) spider. There are also species-specific responses (rats do not spray predators; while skunks do so with glands, and humans with cans of insecticide) but these trigger and effector differences are superficial. Prefrontal and cingulate cortex simply add the capacity for more complex, e.g., social, stimuli to generate clinically problematic panic that is nonetheless primitive. The fundamental internal reactions and control are much the same across all these species; with panicolytic drugs having the same functional effect, including with human social anxiety and obsessive-compulsive disorder (De Oliveira Sergio et al., 2020; Piccinelli, Pini, Bellantuono, & Wilkinson, 1995).

But when reflexive survival circuits or habitual reactions are insufficient to maintain goal-directed behaviour, they must be stopped, and more complex prefrontal control put in their place. For example, anterior cingulate cortex overcomes reflexive action generation and allocation of attention via processes that can be measured in simple behavioural tasks such as the stop-signal (SST), go/no-go, Stroop, and Eriksen flanker (Shackman et al., 2011). The SST is the simplest, designed to assess pure stopping (Logan, Cowan, & Davis, 1984). This simple “ability to suppress unwanted or inappropriate actions and impulses (‘response inhibition’) is a crucial component of flexible and goal-directed behavior … Its derailment is considered integral to numerous neurological and psychiatric disorders, and more generally, to a wide range of behavioral and health problems.” (Verbruggen et al., 2019, p. 2 … p. 1).

The neural basis of stopping is well studied and involves, in particular, the right inferior frontal gyrus in humans – homologous to the orbital frontal area in rats (Aron, Robbins, & Poldrack, 2014). Interestingly, in humans in the SST, a distinct goal-conflict-related right frontal activation (Shadli, Glue, McIntosh, & McNaughton, 2015; Shadli et al., 2020) is a biomarker for anxiety disorder (Shadli et al., 2021) and is inversely linked to Attention Deficit Hyperactivity Disorder (ADHD; Sadeghi et al., 2018). In rats in the SST (a case of inverse translation), the same goal conflict activation involves homologous circuitry including the orbital frontal area, hippocampus, and subthalamus (Banstola, Young, Parr-Brownlie, & McNaughton, 2022). Thus, in stimulus terms, *why* a human chooses to stop differs across occasions and may differ from why a rat does; but *how* rats and humans stop appears to be the same; and stop-go conflict engages homologous parallel circuits that are involved in trait psychopathologies that are occasion-general.

Likewise, rat models of ADHD (with attentional and inhibition deficits across multiple tests) have elucidated dopaminergic and noradrenergic mechanisms (Bayless, Perez, & Daniel, 2015; Li et al., 2021; Russell, Allie, & Wiggins, 2000; Sable et al., 2021). Comparison of two such models (the Spontaneously Hypertensive rat and the New Zealand Genetically Hypertensive rat) in a modified child delayed reinforcement “marshmallow” test allowed a nuanced test of the likely control of immediate reinforcement in ADHD (Sutherland et al., 2009).

Cortical involvement is not all top-down. Emotions impact cognitive control. Emotional stimuli disrupt inhibition in humans (Kalanthroff, Cohen, & Henik, 2013; Pessoa, Padmala, Kenzer, & Bauer, 2012) and non-humans (Kambali, Anshu, Kutty, Muddashetty, & Laxmi, 2019; Klein et al., 2014; Weimar et al., 2020) and disrupt working memory (Bishop, 2007; Bishop & Forster, 2013; Etkin, 2012; Etkin, Gyurak, & O'Hara, 2013; Okon-Singer et al., 2014; van Ast et al., 2016). Emotionally relevant distractors impact task performance via (1) increased activity of ventral brain structures associated with emotional processing, such as the amygdala and ventral prefrontal cortex and (2) decreased activity of dorsal regions involved with executive processing, such as the dorsolateral prefrontal cortex and lateral parietal cortex (Iordan, Dolcos, & Dolcos, 2013). Emotional recovery, neuroticism, and chronic stress are intermingled and associated with disruption in these systems (Blackford, Avery, Shelton, & Zald, 2009; Lapate et al., 2014; Schuyler et al., 2014).

Again, non-human translational models are available. For example, Yee, Leng, Shenhav and Braver (2022) showed how the manipulation of reward and punishment in different rodent models of conditioning tasks may demonstrate whether the presence of the aversive stimulus strengthens or weakens behaviour. Similar tasks in primates confirmed the importance of different regions of the frontal, parietal, and cingulate cortex (Amemori, Amemori, & Graybiel, 2015; Amemori & Graybiel, 2012; Leathers & Olson, 2012) in emotional-motivated decision making (Roesch & Olson, 2004).

Despite all these homologies, there are likely to be some who question the idea of non-human cognition, in and of itself. We have argued against the

“claim that emotion and personality, nonetheless, remain distinct from the rest of biology; that with them it is still the case that ‘the only proper study of mankind is man’ … [with] the role of ‘pure cognition’ as so central to human psychology as to make biology irrelevant, or at least a second-best level of analysis. … We suspect that [a range of essentially anthropocentric] fallacies underlie the separation of biological and cognitive constructs in psychology in general and personality psychology in particular. … The [counter] arguments adhere to two fundamental beliefs in biology: the continuity of species implied by Darwinian evolution; and the mapping of mind to brain as different levels of description of the same fundamental entity. Mind is not here identical to brain. It is a property of brain *processes*. … The human species is, of course, unique. … But no character sets us apart from other animals in a way that other characters do not set each species apart from all others.” (McNaughton & Corr, 2008, pp. 95–101)

There are strong reasons, here, to reject radical behaviourism, primary anthropocentrism, cognitive anthropocentrism; and the ideas that cognitions are: language-dependent; emotionally neutral; unconstrained; hardware-free; silent; and seated in the cortex (McNaughton & Corr, 2008).

## 5. Personality and psychopathology

Latzman, Krueger, DeYoung and Michelini (2021) describe distinct approaches to personality and psychopathology. Personality is viewed dimensionally; but psychopathology is often viewed categorically. However, critical limitations exist in the categorical models of psychopathology (Cuthbert, 2015; Krueger et al., 2018). Instead, empirical evidence favours continuous/dimensional perspectives, such as the MMPI/Minnesota Multiphasic Personality Inventory (Ben-Porath & Tellegen, 2008/2011). “The MMPI has evolved from an innovation that was developed via state-of-the-art procedures in the 1930s into the current MMPI-2-RF that is psychometrically up to date and aligns well with contemporary models of psychopathology. … The MMPI-2-RF substantive scales operationalize psychological constructs that are dimensional and transdiagnostic in nature. The MMPI-2-RF scales map onto the promising HiTOP model, which represents a recent, comprehensive effort to organize psychopathology in a hierarchical and dimensional manner” (Sellbom, 2019, p. 169–170). HiTOP/The Hierarchical Taxonomy of Psychopathology initiative “constructs psychopathological syndromes and their components/subtypes based on the observed covariation of symptoms, [and] combines co-occurring syndromes into spectra, thereby mapping out comorbidity” (Kotov et al., 2017, 2021). Thus, “quantitatively derived, integrative models of personality–psychopathology represent a particularly promising conduit for advancing our understanding of the neurobiological foundation of human experience, both functional and dysfunctional” (Latzman et al., 2021, p. 1).

According to Widiger (2011), the relationship of personality and psychopathology can be approached in three different ways: (1) personality and psychopathology can influence the presentation or appearance of one another; (2) they can share a common, underlying aetiology; or (3) they can have a causal role in the development or aetiology of one another. Empirical evidence in support of the first approach shows, for example, the presence of personality traits of perfectionism and compulsivity in persons with anorexia and impulsivity in those with bulimic symptomatology (Cassin & von Ranson, 2005). On the other hand, while persons high in neuroticism will respond to stress with clinically significant levels of depression, this following depression would lead the patients to provide a distorted description of their usual way of thinking, feeling, behaving, and relating to others, i.e., dimensions of personality (Gunderson et al., 2003). This change in self-report following a mood disorder can be argued to pose as an actual change in personality (Costa, Bagby, Herbst, & McCrae, 2005; Widiger, 2011).

The difficulty of isolating or manipulating the relevant variables in human studies limits understanding of the relationship between personality and psychopathology. Non-human models, then, provide a tool for the analysis of the various genetic, environmental, or pharmacological influences underlying the behavioural expression and physiological functions homologous in non-humans and humans (Kumar, Bhat, & Kumar, 2013). Based on face, predictive, and construct validities, these models have contributed to elucidating different aspects of various psychiatric disorders, such as anxiety, depression, and PTSD (Abelaira, Réus, & Quevedo, 2013; Buenhombre, Daza-Cardona, Sousa, & Gouveia, 2021; Campos, Fogaça, Aguiar, & Guimarães, 2013; Dunsmoor, Cisler, Fonzo, Creech, & Nemeroff, 2022; Gomes Vitor de Castro et al., 2013), autism (Chadman, 2017; Varghese et al., 2017), compulsive eating (Di Segni, Patrono, Patella, Puglisi-Allegra, & Ventura, 2014; Turton, Chami, & Treasure, 2017), and schizophrenia (Jones, Watson, & Fone, 2011; Winship et al., 2018).

## 6. Conclusions

In sum, there is good reason to see non-human models as providing a range of “reduced” examples of the fundamental neural control of emotional (and other psychological) traits. Importantly, these fundamental systems are highly conserved functionally and neurally; with neocortical expansion simply adding superficial complexity to their trigger stimuli and effector outputs. Selection of non-human strains provides an experimental means to answer questions about genes, environment, and their interactions in shaping personality. Non-human models also clearly apply to cognitive as well as emotional traits provided care is taken to determine the relevant neural and behavioural homologies. Finally, in an era where personality and psychopathology are moving to a common integration, non-human models (particularly of psychiatric disorders) provide a means of mapping out the neural bedrock that must be common to both healthy and disordered personality.

We hope this Special Issue will help convince those who construct human personality questionnaires to look to non-human work (particularly neuroscience) as a basis for both construction and validation. There will also always be those who think that their non-human studies cannot, even should not, be used to develop human personality questionnaires. But our main goal is human personality psychology! This raises the issue of how we translate between animal experimental studies and human personality questionnaires.

Such translation is not a new idea. For example, Eysenck’s early human work led to development of the Maudsley rat strains as a model of emotionality or neuroticism (Blizard & Adams, 2002). In the reverse direction, non-human work, via the idea of a Conceptual Nervous System (Gray, 1972a; Hebb, 1955), provided the impetus for the Reinforcement Sensitivity Theory of Personality (Corr, 2008; Gray, 1972b).

But we suggest that such translational work can be deeper. Only a partial connection with non-human and neural bedrock was made, primarily at the scale-construction stage, in development of RST scales (Carver & White, 1994; Wilson, Barrett, & Gray, 1989; Wilson, Gray, & Barrett, 1990), Affective Neuroscience Personality Scales (Davis & Panksepp, 2018; Davis, Panksepp, & Normansell, 2003; Montag, Elhai, & Davis, 2021), and the Tridimensional Personality Questionnaire (Cloninger, 1987; Cloninger, Przybeck, & Svrakic, 1991). These scales used fundamental neurobiology for the theoretical model stage of scale development but used conventional item pool generation and structural validation (Clark & Watson, 2019) to generate linguistically complex items. In some cases, this led to malleable constructs – PANIC in one version of the Affective Neuroscience Personality Scales was later changed to SADNESS on purely semantic grounds (Davis et al., 2003). However, such questionnaires (derived from and interpreted through non-human data) have seldom been directly validated against homologous behavioural or neural measures to those of the original base theories.

With modern developments in genetics, imaging, and translational biomarker development (Shadli et al., 2021), there is now scope for deeper connections to be made and for questionnaire constructs to be validated via neurobiology. Tests using virtual worlds with real-world consequences can link human trait measures to essentially the same behaviours as those measured in non-human tests (Bach et al., 2014; Fung, Qi, Hassabis, Daw, & Mobbs, 2019; Korn & Bach, 2019). Importantly, imaging in these virtual world human tests demonstrates essentially the same neural architecture as detailed in a mass of previous non-human work (McNaughton, 2019). We expect the papers in this Special Issue to open up many such avenues, with traffic flowing in both directions.

## Footnotes

1 Guidelines and a style example can be obtained by emailing the authors of this introduction – to whom a title and brief abstract should then be sent as a presubmission enquiry in order to receive a formal invitation for submission.

2 Fish dorsal telencephalon is considered a homologue of the mammalian amygdala (Jesuthasan, 2012; Lal et al., 2018; Yamaguchi, Danjo, Pastan, Hikida, & Nakanishi, 2013).
